# Resource Allocation Optimization in IoT-Enabled Water Quality Monitoring Systems

**DOI:** 10.3390/s23218963

**Published:** 2023-11-03

**Authors:** Segun O. Olatinwo, Trudi H. Joubert

**Affiliations:** Department of Electrical, Electronic and Computer Engineering, University of Pretoria, Pretoria 0002, South Africa; trudi.joubert@up.ac.za

**Keywords:** water network, water quality monitoring, water quality, water resource management, network resource management

## Abstract

Water quality monitoring systems that are enabled by the Internet of Things (IoT) and used in water applications to collect and transmit water data to data processing centers are often resource-constrained in terms of power, bandwidth, and computation resources. These limitations typically impact their performance in practice and often result in forwarding their data to remote stations where the collected water data are processed to predict the status of water quality, because of their limited computation resources. This often negates the goal of effectively monitoring the changes in water quality in a real-time manner. Consequently, this study proposes a new resource allocation method to optimize the available power and time resources as well as dynamically allocate hybrid access points (HAPs) to water quality sensors to improve the energy efficiency and data throughput of the system. The proposed system is also integrated with edge computing to enable data processing at the water site to guarantee real-time monitoring of any changes in water quality and ensure timely access to clean water by the public. The proposed method is compared with a related method to validate the system performance. The proposed system outperforms the existing system and performs well in different simulation experiments. The proposed method improved the baseline method by approximately 12.65% and 16.49% for two different configurations, demonstrating its effectiveness in improving the energy efficiency of a water quality monitoring system.

## 1. Introduction

Water is an essential resource to humanity [1,2,3,4,5]. Water is life as the human body is made up of approximately 65% water [6]. Hence, humans need to consume clean water for their survival [4,5,7]. Clean water also plays a critical role in improving people’s health, well-being, and quality of life [1,2,5]. However, clean water is becoming a scarce commodity because of the negative impacts of several anthropogenic activities [1,5,7,8,9,10].

To increase access to clean water, water quality monitoring is an active area of research in academia and industry [5,11,12,13]. Water quality monitoring research involves the use of traditional laboratory-based technology as well as modern distributed Internet of Things (IoT) technology. Among these technologies, the use of IoT technology is more popular because of its advantages over laboratory-based technology in terms of cost, real-time water quality monitoring, and prompt water data collection [14,15,16,17]

Despite the promise of IoT systems in water quality monitoring applications, they are still at an infant stage and are resource-constrained in terms of power, bandwidth, and computational resources [18]. Because of these constraints, the IoT systems in water quality monitoring applications are currently limited in performance in the context of energy efficiency, throughput, and network lifetime [19].

For IoT systems to perform better in the water quality monitoring domain, it is important to address the resource constraint issues. Hence, it is important to investigate the design of new resource management strategies that can be incorporated into IoT systems for water quality monitoring. This study focuses on the design of efficient resource allocation strategies for resource management in a non-orthogonal multiple access (NOMA) IoT network for water quality monitoring; the main contributions of this work are highlighted as follows:

- We propose the design of a NOMA-enabled protocol for IoT-enabled water quality monitoring systems;
- We propose the integration of edge computing with water quality monitoring systems;
- We propose resource allocation optimization methods, including a Dinkelbach algorithm-based optimization method for optimizing wireless energy transfer and wireless information transfer, as well as a dynamic resource allocation method for hybrid access point (HAP) resource allocation for data collection;
- We provide a comparison of the proposed method with a comparable baseline method.

The content of this article is organized as follows. In Section 2, we provide a review of the related studies. Section 3 presents the proposed method. Section 4 presents the process of mathematical modeling and optimization of resource allocation. In Section 5, we provide experimental results to illustrate the energy efficiency performance of the proposed NOMA-enabled IoT system for water quality monitoring based on the proposed efficient resource allocation strategies. Section 6 concludes this work.

## 2. Related Work

The successful deployment of IoT systems in water quality monitoring applications require efficient resource allocation solutions that can increase the system energy efficiency and network lifetime, support the transmission of a large amount of water data, and reduce the computational resources required by the system. As a consequence of this, researchers have intensified efforts to propose various resource allocation solutions. A review of the examples of the related studies are presented as follows.

Ji and Guo [20] considered the problem of resource allocation optimization in a wireless-powered mobile edge computing-based IoT network. In their work, the mobile edge computing approach was employed to offload intensive computation tasks from the network devices to the edge nodes because of the resource-constrained nature of IoT devices. However, the use of mobile edge computing in IoT systems often results in increased resource utilization cost (e.g., power) and computational complexity. This may be due to the extra computational overhead and energy consumption introduced by edge computing for sensor devices because they must perform complex tasks in the context of data collection, processing, analysis, and decision making. In addition, the mobile edge computing approach is still a developing technology associated with various resource-allocation problems. Similar to the work of Ji and Guo [20], Ahmed et al. [21], and Sun et al. [22] also considered the problem of resource allocation in wireless-powered mobile edge computing networks. However, these works also suffer from the inherent resource wastage issue associated with edge computing due to the extra computational overhead and energy consumption introduced by edge computing for sensor devices. To address these issues, we introduced a heterogeneous multiclass communication system that consists of ordinary water quality sensor devices and two edge computing-enabled HAP devices that can perform edge computing tasks [23,24]. Additionally, we introduced the concept of a sequential multi-class WPCN to optimally and sequentially schedule the operation of network devices for data transmission with a low-complexity dynamic resource allocation method.

Zeng [25] formulated the system energy efficiency problem as a game theory problem. In their work, the sensor devices are modeled to collectively work together to maximize their overall objective function value. However, because of the need to cooperatively make decisions on how to allocate resources, the system suffered from a computational complexity issue. This resulted in a low energy efficiency performance of the system. To address the problem in the work of Zeng [25], we propose a dynamic resource allocation method and an optimization-based method to jointly optimize the allocation of the system resources and improve the overall system energy efficiency in a sequential multi-class WPCN setting.

Olatinwo and Joubert [26] studied the energy efficiency optimization problem in a wireless-powered sensor network where all the sensor devices in the network only have the opportunity to send their data to only one hybrid access point (HAP). In this case, more energy is spent by the IoT devices that are far from the HAP while less energy is used to send data by the IoT devices that are close to the HAP. This situation is due to the inherent doubly near–far problem in wireless-powered communication networks (WPCNs). To deal with this problem, a WPCN was proposed with two HAPs and a dynamic resource allocation method to efficiently allocate the HAPs to the network IoT devices for their data collection. In addition, unlike the work of Olatinwo and Joubert [26], where a meta-heuristic method was used to compute resource allocation to the network IoT devices with a near-optimal best effort solution, this study considered the application of the Dinkelbach algorithm to compute an optimal solution for the IoT devices in the proposed system.

Ansere et al. [27] studied the problem of resource allocation in a cooperative IoT network for energy efficiency performance optimization of the network. In the network in the work of Ansere et al. [27], a cooperative relaying communication approach is employed to enable the network devices to collectively agree to select a channel (i.e., a relaying link) to send their data to a base station device at the destination. The cooperative communication process between the devices in the study of Ansere et al. [27] for decision making regarding channel selection will technically increase the computational complexity of the network in the context of power and time. This often leads to an increased energy consumption and low data throughput. The concept of cooperative communication in IoT networks is useful for reducing energy consumption due to data communication and increases the amount of data that the network can transmit. However, cooperative communication networks often experience an increased energy consumption and a reduced data throughput under an imperfect channel state. To address the limitations in the study of Ansere et al. [27] and also cater for limited power resources in a cooperative IoT network, this study introduced the use of a wireless power transfer technique to increase the availability of power resources in the network. Furthermore, the two HAPs are used to reduce the energy consumption due to data communication in a quasi-static environment. In addition, the concept of a sequential multi-class WPCN is proposed to optimally and sequentially schedule the operation of the network devices in the uplink using a low complexity dynamic resource allocation method.

Ji et al. [28] studied the problem of resource allocation in wireless-powered IoT networks. In their work, a dual-hop communication approach was employed. In this case, relay node was used as an intermediate node between a power source and the IoT devices. The relay node used the energy received from the power source to power the IoT devices as well as collect data from the IoT devices. This can lead to network congestion and reduced throughput. Furthermore, in a wireless-powered network with one power source, the IoT devices experience unfairness in energy harvesting and data transmission. To address the problems associated with the use of relay nodes in networks powered by a single transmitter, we considered multiple transmitters in this work to improve the energy harvesting. In addition, we introduced two HAPs and a dynamic resource allocation method to optimally allocate the HAPs to the IoT devices for their data collection.

We present a summary of the comparison of the proposed work and the existing works in Table 1.

It is important to emphasize that energy efficiency is still a major concern in IoT systems for several reasons, including the limited power resources of sensor devices and energy consumption due to data communication. Therefore, more research is needed to investigate the design of new resource allocation solutions for IoT systems in practical applications. Based on this, in contrast to the previous studies discussed above, we propose the development of a Dinkelbach algorithm-based method and a dynamic resource allocation method to achieve optimal energy efficiency in the proposed NOMA-enabled IoT system for water quality monitoring.

## 3. Proposed Method

### 3.1. System Architecture

**Figure 1:**
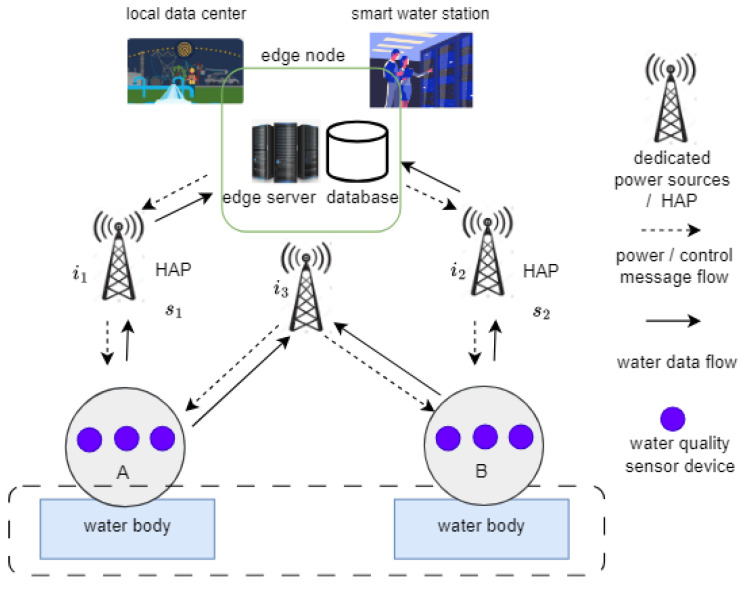
Proposed architecture for water quality monitoring system.

The proposed architecture of the water quality monitoring system is illustrated in Figure 1. It consists of a set of water quality sensors, HAPs, and an edge computing node. To provide readers with more insights into the project, Table 2 shows a list of requirements for the system architecture. The water quality sensors are deployed at a water site to capture the water quality parameters of the relevant water body. Some of the important parameters for drinking water include pH, E. coli, and dissolved oxygen [29,30,31,32]. Due to the power-constrained nature of water quality sensors in IoT systems, HAPs are employed to power sensor devices and collect water data from sensor devices. The edge computing node is introduced to increase the computational capacity of the system for local water data processing. This is motivated by the limited computational resources of sensor devices and the gap in conventional water quality monitoring systems. For example, in most water quality monitoring systems, water data are often forwarded to remote stations where data processing, analysis, and prediction take place. By sending water data to distant remote water stations, real-time monitoring of any possible changes in water parameters may be impractical. To address this gap, we combined edge computing with the proposed water quality monitoring system to enable real-time water quality monitoring.

### 3.2. System Model

This study considers a multi-class communication system that classifies the system sensor devices into two different classes, A and B, according to the channel gains among the sensor devices and the data collecting HAPs. To minimize the transmission power used by the sensor devices to transfer their individual data to the available HAPs, the proposed system exploits the channel gain differences among the sensor devices and the available HAPs in the system to classify them into *K* sensor devices ∈ $m=\{m_{1},m_{2},\ldots ,m_{K}\}$ and *L* sensor devices ∈ $n=\{n_{1},n_{2},\ldots ,n_{L}\}$.

In the system, there are *I* dedicated power sources, represented as $s=\{s_{1},s_{2},\ldots ,s_{I}\}$. These power sources are used to transfer power to *K* sensor devices in class *A* as well as the *L* sensor devices in class *B*. Among the *I* power sources, two of them ($s_{1}$ and $s_{2}$) serve as the HAPs. Hence, both $s_{1}$ and $s_{2}$ ∈ *s* can transfer power to the sensor devices and can also collect data from the sensor devices. Each class of the network transfers its data to two HAPs to achieve a good channel gain among the sensor devices and the HAPs. We assume that the proposed multi-class communication system is a heterogeneous system such that the sensor devices are ordinary water quality sensor devices and HAPs $s_{1}$ and $s_{2}$ are edge computing-enabled and can perform edge computing tasks [23,24]. This circumvents the potential extra computational overhead and energy consumption that edge computing may introduce for sensor devices owing to complex tasks relating to data collection, processing, analysis, and decision making.

**Figure 2:**
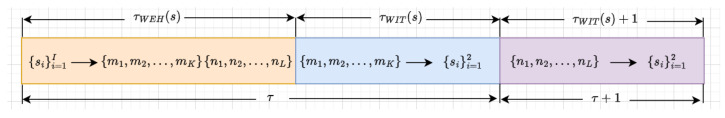
Proposed NOMA-based communication protocol.

The proposed multi-class communication system works as a wireless-powered communication network (WPCN). Hence, the wireless energy harvesting (WEH) phase and the wireless information transmission (WIT) phase of the system operates within a timeslot defined by τ(s) based on the proposed communication protocol presented in Figure 2. Consequently, the durations of the WEH and WIT phases are defined as $\tau_{WEH}\left(s\right)$ and $\tau_{WIT}\left(s\right)$, respectively. Hence, the system operates within the duration of $\tau =\tau_{WEH}\left(s\right)+\tau_{WIT}\left(s\right)$.

In each timeslot, both classes A and B harvest power from all the available *I* power sources within the duration of $\tau_{WEH}\left(s\right)$, whereas only one of the classes is enabled to perform data transmission to the allocated set of HAPs within the duration of $\tau_{WIT}\left(s\right)$. For example, if class A is enabled for data transmission to the HAPs in timeslot τ then class B is sequentially enabled for data transmission to the HAPs in the next timeslot, τ+1. Since it is not a must for all the sensor devices to perform data transmission in the WIT phase concurrently in each timeslot, the concept of sequential data transmission scheduling is considered in this work to optimize the use of power resources by the overall system.

The *K* and *L* sensor devices are strategically deployed across the water body in a random manner to optimally capture the key water parameters, as shown in Figure 1. In addition, dedicated *I* power sources, including the HAPs ${\left\{s_{i}\right\}}_{i=1}^{2}$, are connected to a controller with global knowledge of the resources in the proposed system. It is aware of a scheduler designed to enable either class A or B in a sequential manner for data transmission to HAPs $s_{1}$ and $s_{2}$ in each timeslot, τ. It is important to emphasize that, at each timeslot, the controller update its information about the *K* sensor devices in class *A*, *L* sensor devices in class *B*, and *I* power sources for the purpose of synchronization. The sensor devices draw energy from separate batteries for updating their energy status information with the controller.

Table 3 contains some of key acronyms used in this work.

## 4. Mathematical Model

The communication channel gains among the *K* sensor devices and the HAPs, $s_{1}$ and $s_{2}$, as well as the *L* sensor devices and the HAPs, follow a quasi-static fading model. Therefore, the communication channel gain from the *I* power sources to the *K* sensor devices and the *L* sensor devices in the WEH phase are $c_{i,k}$ and $c_{i,l}$. In addition, the reversed communication channel gain from the *K* sensor devices to the HAPs is $d_{k,i}$, while the communication channel gain from the *L* sensor devices to the HAPs is $d_{l,i}$.

As a result of the reciprocity of DL and UL communication channel gains for the class A network, $c_{i,k}=d_{k,i}=10^{-1}\times d_{k,i}^{-\alpha }$. Furthermore, the reciprocity of the downlink (DL) and uplink (UL) communication channel gains for the class B network is $c_{i,l}=d_{l,i}=10^{-1}\times d_{l,i}^{-\alpha }$. In both cases, α is the pathloss exponent. During the WEH phase, using the proposed NOMA protocol, the *K* sensor devices and the *L* sensor devices harvest energy within the duration of $0\leq \tau_{WEH}\leq 1$. The energy harvested by the *K* sensor devices and the *L* sensor devices are $e_{k\cdot harvest}^{\tau }\left(J\right)$ and $e_{l\cdot harvest}^{\tau }\left(J\right)$, respectively, where:

(1) $$e_{k\cdot harvest}^{\tau }=\xi \sum_{i=1}^{I}\;P_{i}c_{i,k}\tau_{WEH}+e_{k\cdot available}^{\tau },\forall k\;\left(J\right)$$

(2) $$e_{l\cdot harvest}^{\tau }=\xi \sum_{i=1}^{I}\;P_{i}c_{i,l}\tau_{WEH}+e_{l\cdot available}^{\tau },\forall l\;\left(J\right)$$

where $P_{i}\left(W\right)$ is the transmission power used by the *I* power sources to charge the *K* sensor devices, while $e_{k\cdot harvest}^{\tau }$ and $e_{l\cdot harvest}^{\tau }$ are the available energy in the *K* and *L* sensor devices’ in-built batteries from the previous timeslot.

Each *k* sensor device and *l* sensor device used the energy $e_{k\cdot transmit}^{\tau }\;\left(J\right)$ and $e_{l\cdot transmit}^{\tau }\;\left(J\right)$ to transfer their respective data to HAPs $s_{1}$ and $s_{2}$, respectively, in the WIT phase. Consequently, the available energy, $e_{k\cdot available}^{\tau }$ and $e_{l\cdot available}^{\tau }$, for the next timeslot, τ+1, is computed based on (3) and (4):

(3) $$e_{k\cdot available}^{\left(\tau +1\right)}=e_{k\cdot harvest}^{\tau }-e_{k\cdot transmit}^{\tau }$$

(4) $$e_{l\cdot available}^{\left(\tau +1\right)}=e_{l\cdot harvest}^{\tau }-e_{l\cdot transmit}^{\tau }$$

The transmission power used by each *k* sensor device to transfer its data to HAPs $s_{1}$ and $s_{2}$ is defined as $P_{k,i}\left(W\right)$, while $P_{l,i}\left(W\right)$ is the transmission power used by each *l* sensor device to transmit its data to the HAPs. During the WIT phase, using the NOMA protocol, the *K* sensor devices and *L* sensor devices transmit their individual data at a scheduled timeslot, τ, to their dynamically allocated HAPs, ${\left\{s_{i}\right\}}_{i=1}^{2}$, within the duration of $0\leq \tau_{WIT}\leq 1$.

Because of the simultaneous data transmission of the *K* sensor devices and the *L* sensor devices at a scheduled timeslot to the HAPs, a successive interference cancellation (SIC) technique is applied at the HAPs to enable a sequential decoding of the concurrently transmitted data of the *K* sensor devices as well as the *L* sensor devices in each timeslot by first decoding the signal of the highest channel gain sensor device at the corresponding HAP *i* [26,33].

By applying the Shannon theory, the amount of data that each *k* sensor device can transmit per second to a HAP *i* in the WIT phase is computed in (5) as:

(5) $$\begin{array}{cc} R_{k,i}\left(\tau ,P_{k,i}\right) & =\sum_{i\in s}\tau_{WEH}Blog_{2}\\ & \left(1+\frac{P_{k,i}d_{k,i}}{\sum_{k^{\prime }\geq k+1}P_{k^{\prime },i}d_{k^{\prime },i}+\sigma^{2}}\right)\\ & \forall k\in \{m_{1},m_{2},\ldots ,m_{K}\} \end{array}$$

where *B* denotes the system’s bandwidth in Hz, $d_{k,i}$ denotes the UL communication channel from the *K* sensor devices to the HAPs, $P_{k,i}\left(W\right)$ represents the transmission power consumed by each *k* sensor device to send its data to a HAP *i*, and $\sigma^{2}$ is the additive white Gaussian noise (AWGN) power. The resource allocation vectors for the time resources and the transmission power resources for the *K* sensor devices are formulated as $\tau ={\left[\tau_{WEH},\tau_{WIT}\right]}^{T}$ and $P_{A}={\left[P_{1,1},P_{2,1},P_{3,1},\ldots ,P_{K,1},P_{1,2},P_{2,2},P_{3,2},\ldots ,P_{K,2}\right]}^{T}$, respectively.

A minimum quality of service (QoS) constraint is set for each sensor device *k* in (6) to satisfy the minimum amount of data of the *K* sensor devices in order to achieve a reliable data transmission.

(6) $$R_{k,i}\left(\tau ,P_{k,i}\right)\geq r_{k,i},\forall k$$

The total amount of data that all the *K* sensor devices can transmit is computed in (7) from (5) as:

(7) $$R_{total}\left(\tau ,P_{k,i}\right)=\sum_{k=1}^{K}\sum_{i\in s}R_{k,i}\left(\tau ,P_{k,i}\right),\forall k$$

Furthermore, the amount of data that each *l* device can transmit per second to a HAP *i* during the WIT phase is formulated in (8) as:

(8) $$\begin{array}{cc} R_{l,i}\left(\tau ,P_{l,i}\right) & =\sum_{i\in s}\tau_{WEH}Blog_{2}\\ & \left(1+\frac{P_{l,i}d_{l,i}}{\sum_{l^{\prime }\geq l+1}P_{l^{\prime },i}d_{l^{\prime },i}+\sigma^{2}}\right)\\ & \forall l\in \{n_{1},n_{2},\ldots ,n_{L}\} \end{array}$$

The resource allocation vectors for the time resources and the transmission power resources for the *L* sensor devices are $\tau ={\left[\tau_{WEH},\tau_{WIT}\right]}^{T}$ and $P_{B}=[P_{1,1},P_{2,1},P_{3,1},\ldots ,P_{L,1},P_{1,2}$, $P_{2,2},P_{3,2},\ldots ,P_{L,2}{]}^{T}$, respectively. Additionally, the minimum QoS rate constraint for the *L* sensor devices is formulated in (9) as:

(9) $$R_{l,i}\left(\tau ,P_{l,i}\right)\geq r_{l,i},\forall i$$

The total amount of data that all the *L* sensor devices can transmit is calculated in (10) as:

(10) $$R_{total}\left(\tau ,P_{l,i}\right)=\sum_{l=1}^{L}\sum_{i\in s}R_{l,i}\left(\tau ,P_{l,i}\right),\forall l$$

During the WEH phase, the energy consumed by the *K* devices and *L* devices are computed in (11) and (12) [26] as:

(11) $$\begin{array}{cc} e_{K,WEH}\left(\tau_{WEH},P_{i}\right) & =\sum_{i=1}^{I}\left(P_{i}+P_{c}-\sum_{k=1}^{K}\xi \left(c_{i,k}P_{i}\right)\right)\tau_{WEH},\;\forall k \end{array}$$

where $P_{c}$ is the circuit power consumption for the transmission power and hardware.

(12) $$\begin{array}{cc} e_{L,WEH}\left(\tau_{WEH},P_{i}\right) & =\sum_{i=1}^{I}\left(P_{i}+P_{c}-\sum_{l=1}^{L}\xi \left(c_{i,l}P_{i}\right)\right)\tau_{WEH},\;\forall l \end{array}$$

By combining (11) and (12), the total energy consumed by both *K* devices and *L* devices during the WEH phase is formulated in (13) as:

(13) $$\begin{array}{cc} e_{WEH}^{K,L}\left(\tau_{WEH},P_{i}\right) & =\sum_{i=1}^{I}\left(P_{i}+P_{c}-\sum_{k=1}^{K}\xi \left(c_{i,k}P_{i}\right)+\sum_{l=1}^{L}\xi \left(c_{i,l}P_{i}\right)\right)\tau_{WEH} \end{array}$$

During the WIT phase, the energy consumed by the *K* devices and *L* devices to transmit their individual data to an allocated HAP *i* at a scheduled period is formulated in (14) and (15) as:

(14) $$e_{K\cdot WIT}\left(\tau_{WIT},P_{k,i}\right)=\sum_{k=1}^{K}\left(P_{k,i}+P_{k,c}\right)\tau_{WIT}$$

where $P_{k,c}$ is the circuit power consumption for the *k* device.

(15) $$e_{L\cdot WIT}\left(\tau_{WIT},P_{l,i}\right)=\sum_{l=1}^{L}\left(P_{l,i}+P_{l,c}\right)\tau_{WIT}$$

where $P_{l,c}$ is the circuit power consumption for the *l* device.

Based on the derived equations, the total energy consumed by the class A devices at a timeslot, τ, and the total energy consumed by the class B devices at the next time timeslot, τ+1, can now be formulated in (16)–(19).

(16) $$e_{K\cdot total}=e_{WEH}^{K,L}\left(\tau_{WEH},P_{i}\right)+e_{K\cdot WIT}\left(\tau_{WIT},P_{k,i}\right)$$

(17) $$\begin{array}{cc} e_{K\cdot total}\left(\tau_{WEH},P_{i},\tau_{WIT},P_{k,i}\right) & =\sum_{i=1}^{I}\left(P_{i}+P_{c}-\sum_{k=1}^{K}\xi \left(c_{i,k}Pi\right)+\sum_{l=1}^{L}\xi \left(c_{i,l}P_{i}\right)\right)\tau_{WEH}+\\ & \sum_{k=1}^{K}\left(P_{k,i}+P_{k,c}\right)\tau_{WIT} \end{array}$$

(18) $$e_{L\cdot total}=e_{WEH}^{K,L}\left(\tau_{WEH},P_{i}\right)+e_{L\cdot WIT}\left(\tau_{WIT},P_{l,i}\right)$$

(19) $$\begin{array}{cc} e_{L\cdot total}\left(\tau_{WEH},P_{i},\tau_{WIT},P_{l,i}\right) & =\sum_{i=1}^{I}\left(P_{i}+P_{c}-\sum_{k=1}^{K}\xi \left(c_{i,k}Pi\right)+\sum_{l=1}^{L}\xi \left(c_{i,l}P_{i}\right)\right)\tau_{WEH}+\\ & \sum_{l=1}^{L}\left(P_{l,i}+P_{l,c}\right)\tau_{WIT} \end{array}$$

According to [34], the system energy efficiency (EE) is the ratio of the received sum-data and the total power consumption. Due to the scheduling of the *K* devices in class A and the *L* devices in class B during the WIT phase, problems (17) and (19) are solved independently at timeslot τ and the next timeslot, τ+1. Therefore, the system EE configured with *K* sequential devices in class A at timeslot τ is formulated as an optimization problem in (20), and the time allocation, τ, the power source power allocation, $P_{1}$, as well as each *k* device power allocation, $P_{k,i}$, are jointly optimized. The system EE optimization problem is:

(20) $$P1:\underset{\tau ,P_{i},P_{k,i}}{max}\;\frac{R_{total}\left(\tau ,P_{k,i}\right)}{e_{K\cdot total(\tau ,P_{i},P_{k,i})}}$$

s.t.:

(21) $$C1:\tau_{WEH}+\tau_{WIT}\leq 1$$

(22) $$C2:0\leq P_{i}\leq P_{i}^{max}$$

(23) $$C3:0\leq P_{k,i}\leq P_{k,i}^{max}$$

(24) $$C4:\left(P_{k,i}+P_{k,c}\right)\tau_{WIT}\leq \xi \sum_{i=1}^{I}P_{i}c_{i,k}\tau_{WEH}e_{k.available}^{\left(\tau \right)}$$

(25) $$C5:\tau_{WEH}\geq 0,\forall i\cup \forall k$$

(26) $$C6:\tau_{WIT}\geq 0,\forall k\cup {\left\{s_{i}\right\}}_{i=1}^{2}$$

where C1 is the time resource allocation constraint, C2 is the transmission power constraint for the power sources, C3 represents the limit on the sensor device *k* transmission power, the C4 constraint ensures that the power cost for sensor device *k* data transmission should not exceed its total power, and C5 and C6 are non-negative constraints for the decision variables.

In (20), $\tau_{WEH}$ and $\tau_{WIT}$ are replaced with τ in subsequent problems involving $\tau_{WEH}$ and $\tau_{WIT}$ since $\tau =\tau_{WEH}+\tau_{WIT}$.

The system EE configured with *L* sequential devices in class B at the next timeslot, τ+1, is formulated in (27), and the time allocation, τ, the power source power allocation, $P_{i}$, as well as the power allocation, $P_{l,i}$, of each *l* device are jointly optimized. The system EE optimization problem is written as:

(27) $$P1:\underset{\tau ,P_{i},P_{l,i}}{max}\;\frac{R_{total}\left(\tau ,P_{l,i}\right)}{e_{L\cdot total(\tau ,P_{i},P_{l,i})}}$$

s.t.:

C1, C2

(28) $$C7:0\leq P_{l,i}\leq P_{l,i}^{max}$$

(29) $$C8:\left(P_{l,i}+P_{l,c}\right)\tau_{WIT}\leq \xi \sum_{i=1}^{I}P_{i}c_{i,l}\tau_{WEH}e_{l.available}^{\left(\tau \right)}$$

(30) $$C9:\tau_{WEH}\geq 0,\forall i\cup \forall l$$

(31) $$C10:\tau_{WIT}\geq 0,\forall l\cup {\left\{s_{i}\right\}}_{i=1}^{2}$$

### 4.1. Transformation of the Objective Function

The optimization problems in (20) and (27) are non-linear fractional optimization problems. Such optimization problems cannot be easily solved directly and it is difficult to obtain optimal solutions to such problems. To deal with this problem, we applied the Dinkelbach method  [35] to transform the non-linear fractional optimization problems into a subtraction form that can be easily solved.

To apply the Dinkelbach method, we introduced parameters *q* and *r* to compute the optimal solution for the system EE in (20) and (27). Let $q^{*}$ represents the system EE for the optimization problem in (20), which is formulated in (32) as:

(32) $$q^{*}=\underset{\tau ,P_{i},P_{k,i}}{max}\;\frac{R_{total}\left(\tau ,P_{k,i}\right)}{e_{K\cdot total(\tau ,P_{i},P_{k,i})}}=\frac{R_{total}\left(\tau^{*},P_{k,i}^{*}\right)}{e_{K\cdot total(\tau^{*},P_{i}^{*},P_{k,i}^{*})}}$$

From (32), the maximum system EE $q^{*}$ can now be easily obtained  [36] when $\underset{\tau^{,}P_{i},P_{k,i}}{max}R_{total}\left(\tau ,P_{k,i}\right)-q^{*}\cdot e_{K\cdot total}\left(\tau ,P_{i},P_{k,i}\right)=R_{total}\left(\tau^{*},P_{k,i}^{*}\right)-q^{*}\cdot e_{K\cdot total}\left(\tau^{*},P_{i}^{*},P_{k,i}^{*}\right)=0$.

By applying the parameter *q* to the system EE optimization problem in (20), problem P1 was transformed as a new objective function in (33) as:

(33) $$P3:\underset{\tau ,P_{i},P_{k,i}}{max}R_{total}\left(\tau ,P_{k,i}\right)-q\cdot e_{K\cdot total}\left(\tau ,P_{i},P_{k,i}\right)$$

s.t.:

C1, C2, C3, C4, C5, and C6

Let $r^{*}$ represents the system EE for problem P2 in (27), which is formulated in (34) as:

(34) $$r^{*}=\underset{\tau ,P_{i},P_{l,i}}{max}\;\frac{R_{total}\left(\tau ,P_{l,i}\right)}{e_{L\cdot total(\tau ,P_{i},P_{l,i})}}=\frac{R_{total}\left(\tau^{*},P_{l,i}^{*}\right)}{e_{L\cdot total(\tau^{*},P_{i}^{*},P_{l,i}^{*})}}$$

From (34), the maximum system EE $r^{*}$ can now be easily obtained when

$\underset{\tau^{,}P_{i},P_{l,i}}{max}R_{total}\left(\tau ,P_{l,i}\right)-r^{*}\cdot e_{L\cdot total}\left(\tau ,P_{i},P_{l,i}\right)=R_{total}\left(\tau^{*},P_{l,i}^{*}\right)-r^{*}\cdot e_{L\cdot total}\left(\tau^{*},P_{i}^{*},P_{l,i}^{*}\right)=0$.

Following this, the parameter *r* can now be applied to optimization problem P2 in (28) to transform it to a new objective function in (35) as:

(35) $$P4:\underset{\tau ,P_{i},P_{k,i}}{max}R_{total}\left(\tau ,P_{k,i}\right)-q\cdot e_{K\cdot total}\left(\tau ,P_{i},P_{k,i}\right)$$

s.t.:

C1, C2, C7, C8, C9, and C10

The convergence of the transformed subtraction function has been proved in  [36], and this can be easily applied to problems (P3) and (P4). Hence, the proof is omitted in this paper. To achieve an optimal EE for the proposed WPCN system, we solved problems (33) and (35) in each iteration using an iteration algorithm.

### 4.2. Optimal Solution

The objective functions in problems (33) and (35) are convex optimizations with respect to variables $\tau ,P_{i},P_{k,i}$ and variables $\tau ,P_{i},P_{l,i}$, respectively. Hence, we proposed and applied a Lagrangian method and a Dinkelbach iterative algorithm. The Lagrangian function of the optimization problem in (33) is given in (36) as:

(36) $$\begin{array}{cc} L(\tau ,P_{i},P_{k,i},\mu_{1},\mu_{2},\mu_{3},\mu_{4}) & =R_{total}\left(\tau ,P_{k,i}\right)-\\ & q\cdot e_{K\cdot total}\left(\tau ,P_{i},P_{k,i}\right)+\\ & \mu_{1}\left(\tau_{WEH}+\tau_{WIT}-1\right)+\\ & \mu_{2}\left(P_{i}-P_{i}^{m}ax\right)+\\ & \mu_{3}\left(P_{k,i}-P_{k,i}^{m}ax\right)+\\ & \mu_{4}(\left(P_{k,i}+P_{k,c}\right)\tau_{WIT}-\\ & \xi \sum_{i=1}^{I}P_{i}c_{i,k}\tau_{WEH}+e_{k.available}^{\left(\tau \right)}) \end{array}$$

where $\tau =(\tau_{WEH},\tau WIT)$ defines the duration for the WEH phase and the duration for the WIT phase and $\mu =(\mu_{1},\mu_{2},\mu_{3},\mu_{4})$ represents the Lagrangian multipliers for the constraints.

The dual optimization problem for the transformed optimization problem in (33) is provided in (37) as:

(37) $$\underset{\tau ,P_{i},P_{k,i},\mu_{1},\mu_{2},\mu_{3},\mu_{4}}{min}\;max\;L\left(\tau ,P_{i},P_{k,i},\mu_{1},\mu_{2},\mu_{3},\mu_{4}\right)$$

s.t.:

$\mu_{1},\mu_{2},\mu_{3},\mu_{4}\geq 0$

Based on the zero-duality-gap condition, the optimal solution of the dual variables (or multipliers) $\mu_{1}^{*},\mu_{2}^{*},\mu_{3}^{*},\mu_{4}^{*}$ will achieve the maximum EE of the problem in (33). This is computed by using an iteration algorithm to solve (37).

For each iteration, the dual optimization problem in (37) is solved using the Karush–Kuhn–Tucker (KKT) conditions  [37] based on the initial variables $\mu_{1},\mu_{2},\mu_{3},\mu_{4}$, and by equating the Lagrangian partial derivative to zero to obtain optimal resource allocation solutions for $\tau ,P_{i},P_{k,i}$.

The iteration process is provided as follows:

$\Delta \mu_{1}=\tau_{WEH}+\tau_{WIT}-1$

$\Delta \mu_{2}=P_{i}-P_{i}^{max}$

$\Delta \mu_{3}=P_{k,i}-P_{k,i}^{max}$

$\Delta \mu_{4}=\left(P_{k,i}+P_{k,c}\right)\tau_{WIT}-\xi \sum_{i=1}^{I}P_{i}c_{i,k}\tau_{WEH}+e_{k.available}^{\left(\tau \right)}$  

The initial Lagrangian multipliers are updated iteratively by $\mu^{\left(t+1\right)}=\left(\mu^{\left(t\right)}+\beta^{\left(t\right)}\Delta \mu \right)$ to obtain a new set of multipliers. This process is repeated until the optimal multipliers are obtained when the proposed iteration algorithm saturates to convergence. Note that $\Delta \mu =(\Delta \mu_{1},\Delta \mu_{2},\Delta \mu_{3},\Delta \mu_{4})$, $\beta^{\left(t\right)}$ is used to denote the step size of the iteration and *T* is used to denote the number of iterations.

For a given μ, the process of computing optimal energy transfer time allocation; data transfer time allocation; and *k* device transmit power allocation, i.e., $\tau^{*}$, $P_{i}^{*}$, and $P_{k,i}^{*}$, is obtained through the KKT conditions by equating the Lagrangian partial derivative to derivatives to zero as follows:

By equating $\frac{\partial L}{\partial \tau_{WEH}}$, $\frac{\partial L}{\partial \tau_{WIT}}$, $\frac{\partial L}{\partial P_{i}}$, and $\frac{\partial L}{\partial P_{k,i}}$ to zero, the following optimal solutions can be obtained.

(38) $$\begin{array}{cc} \frac{\partial L}{\partial \tau_{WEH}} & =\frac{B\sum_{k=1}^{K}\sum_{i\in s}}{In2}In\left(1+\frac{P_{k,i}d_{k,i}}{\sum_{k^{\prime }\geq k+1}P_{k^{\prime },i}d_{k^{\prime },i}+\sigma^{2}}\right)\\ & -q\cdot \sum_{i=1}^{I}(P_{i}+P_{c}-\sum_{k=1}^{K}\xi \left(c_{i,k}P_{i}\right)\\ & +\sum_{l=1}^{L}\xi \left(c_{i,l}Pi\right))+\mu_{1}\left(WIT\right)\\ & +\mu_{4}(\left(P_{k,i}+P_{k,c}\right)\tau_{WIT}\\ & -\xi \sum_{i=1}^{I}P_{i}c_{i,k}+e_{k.available}^{\left(\tau \right)}) \end{array}$$

(39) $$\begin{array}{cc} \frac{\partial L}{\partial \tau_{WIT}} & =\sum_{k=1}^{K}\left(P_{k,i}+P_{k,c}\right)+\mu_{1}\left(\tau_{WEH}\right)+\mu_{4}(\left(P_{k,i}+P_{k,c}\right) \end{array}$$

(40) $$\begin{array}{cc} \frac{\partial L}{\partial P_{i}} & =q\cdot \sum_{i=1}^{I}\left(1+P_{c}-\sum_{k=1}^{K}\xi \left(c_{i,k}\right)+\sum_{l=1}^{L}\xi \left(c_{i,l}\right)\right)\tau_{WEH}\\ & -\mu_{2}\left(1-P_{i}^{max}\right)+\mu_{4}\left(\xi \sum {i=1}^{I}c_{i,k}+\tau_{WEH}+e_{k\cdot available}^{\left(\tau \right)}\right) \end{array}$$

(41) $$\begin{array}{cc} \frac{\partial L}{\partial P_{k,i}} & =\frac{B\sum_{k=1}^{K}\sum_{i\in s}\tau_{WEH}}{In2}\\ & \left(\frac{\sum_{k^{\prime }\geq k+1}P_{k^{\prime },i}d_{k^{\prime },i}+\sigma^{2}}{\sum_{k^{\prime }\geq k+1}P_{k^{\prime },i}d_{k^{\prime },i}+\sigma^{2}+P_{k,i}d_{k,i}}\right)\cdot \\ & \left(\frac{d_{k,i}}{\sum_{k^{\prime }\geq k+1}P_{k^{\prime },i}d_{k^{\prime },i}+\sigma^{2}}\right)+\\ & \sum_{k=1}^{K}\left(1+P_{k,c}\right)\tau_{WIT}+\mu_{3}\left(1-P_{k,i}^{max}\right)\;+\\ & \mu_{4}\left(1+P_{k,c}\right)\tau_{WIT} \end{array}$$

(42) $$\begin{array}{c} \frac{\partial L}{\partial \mu_{1}}=\tau_{WEH}+\tau_{WIT}-1 \end{array}$$

(43) $$\begin{array}{c} \frac{\partial L}{\partial \mu_{2}}=P_{i}-P_{i}^{max} \end{array}$$

(44) $$\begin{array}{c} \frac{\partial L}{\partial \mu_{3}}=P_{k,i}-P_{k,i}^{max} \end{array}$$

(45) $$\begin{array}{cc} \frac{\partial L}{\partial \mu_{4}} & =(\left(P_{k,i}+P_{k,c}\right)\tau_{WIT}\;-\xi \sum_{i=1}^{I}P_{i}c_{i,k}\tau_{WEH}e_{k\cdot available}^{\left(\tau \right)} \end{array}$$

The resource allocation algorithm for solving the optimization problem in (33) is provided in Algorithm 1. In addition, to obtain optimal resource allocations, a Dinkelbach-based iteration algorithm is proposed and presented in Algorithm 2.
**Algorithm 1** Resource Allocation Algorithm**Require:** 
Variables $\mu_{1}$, $\mu_{2}$, $\mu_{3}$, $\mu_{4}$**Ensure:** 
$\lambda_{1}$, $\lambda_{2}$, $\lambda_{3}$, $\lambda_{4}$  1:**for** each *k* in Class *A* **do**  2:    compute an optimal power resource allocation, $P_{k,i}^{*}$, using (41)  3:    compute an optimal time resource allocation, $\tau_{WEH}^{*}$ and $\tau_{WiT}^{*}$, using (38) and (39)  4:    update $\mu_{1},\mu_{2},\mu_{3},\;\mu_{4}$ until convergence  5:**end for**  6:**for** each *l* in Class *B* **do**  7:    compute an optimal power allocation, $P_{L,i}^{*}$, using (51)  8:    compute an optimal time resource allocation, $\tau_{WiT}^{*}$, using (48) and (49)  9:    update $\lambda_{1},\lambda_{2},\lambda_{3},\lambda_{4}$  until convergence10:**end for**

**Algorithm 2** Proposed Dinkelbach-based Iteration Algorithm
**Require:** 
q=0, r=0, t=0, $\tau_{max}=maximum\;number\;of\;iterations$, $e_{max}\;=\;maximum\;error$,  1:**for** each *k* in Class *A* **do**  2:    repeat  3:    apply Algorithm 2 to obtain $\left\{\tau ,P_{k,i}\right\}$  4:    **if** $R_{total}\;\left(\tau ,P_{k,i}\right)-q_{i}\;e_{K.total}\left(\tau ,P_{i},P_{k,i}\right)\;\leq e_{max}$ **then**  5:        return $\left(\tau^{*},P_{k,i}^{*}\right)$  6:        $q^{*}=\frac{R_{total}\left(\tau^{*}P_{k}^{*},i\right)}{e_{K.total}\left(\tau^{*},P_{i}^{*},P_{k,i}^{*}\right)}$  7:    **else** $q=\frac{R_{total}\left(\tau ,P_{k,i}\right)}{e_{K.total}\left(\tau ,P_{i},P_{k,i}\right)},\;t=t+1$  8:    **end if**  9:    Until $R_{total}\left(\tau ,P_{k,i}\right)-r.e_{K.total}\left(\tau ,P_{i},P_{k,i}\right)\;\leq e_{max}$  is true10:
**end for**
11:**for** each *l* in Class *B* **do**12:    repeat13:    apply Algorithm 2 to compute $\left\{\tau ,P_{l,i}\right\}$14:    **if** $R_{total}\;\left(\tau ,P_{l,i}\right)-r_{i}\;e_{L.total}\left(\tau ,P_{i},P_{l,i}\right)\;\leq e_{max}$ **then**15:        return $\left(\tau^{*},P_{l,i}^{*}\right)$16:        $r^{*}=\frac{R_{total}\left(\tau^{*}P_{l}^{*},i\right)}{e_{L.total}\left(\tau^{*},P_{i}^{*},P_{l,i}^{*}\right)}$17:    **else** $r=\frac{R_{total}\left(\tau ,P_{l,i}\right)}{e_{L.total}\left(\tau ,P_{i},P_{l,i}\right)},\;t=t+1$18:    **end if**19:    Until $R_{total}\left(\tau ,P_{l,i}\right)-r.e_{L.total}\left(\tau ,P_{i},P_{l,i}\right)\;\leq e_{max}$  is true20:
**end for**



The Lagrangian function of the optimization problem in (35) is provided in (46) as:

(46) $$\begin{array}{cc} L(\tau ,P_{i},P_{l,i},\lambda_{1},\lambda_{2},\lambda_{3},\lambda_{4}) & =R_{total}\left(\tau ,P_{l,i}\right)-\\ & r\cdot e_{L\cdot total}\left(\tau ,P_{i},P_{l,i}\right)+\\ & \lambda_{1}\left(\tau_{WEH}+\tau_{WIT}-1\right)+\\ & \lambda_{2}\left(P_{i}-P_{i}^{max}\right)+\\ & \lambda_{3}\left(P_{l,i}-P_{l,i}^{max}\right)+\\ & \lambda_{4}(\left(P_{l,i}+P_{l,c}\right)\tau_{WIT}-\\ & \xi \sum_{i=1}^{I}P_{i}c_{i,l}\tau_{WEH}+e_{l.available}^{\left(\tau \right)}) \end{array}$$

where $\lambda =(\lambda_{1},\lambda_{2},\lambda_{3},\lambda_{4})$ denotes the Lagrangian multipliers for the constraints.

The dual optimization problem for the transformed optimization problem in (35) is provided in (47) as:

(47) $$\underset{\tau ,P_{i},P_{l,i},\lambda_{1},\lambda_{2},\lambda_{3},\lambda_{4}}{min}\;max\;L\left(\tau ,P_{i},P_{l,i},\lambda_{1},\lambda_{2},\lambda_{3},\lambda_{4}\right)$$

s.t.:

$\lambda_{1},\lambda_{2},\lambda_{3},\lambda_{4}\geq 0$

According to the zero-duality-gap condition, the optimal solution of the dual variables or multipliers $\lambda_{1}^{*},\lambda_{2}^{*},\lambda_{3}^{*},\lambda_{4}^{*}$ will achieve the maximum EE of the problem in (35). This is computed by the proposed iteration algorithm to solve (47).

In each iteration, the formulated dual optimization problem in (47) is solved by applying the KKT conditions  [37] according to the given initial multipliers $\lambda_{1},\lambda_{2},\lambda_{3},\lambda_{4}$, and by equating the Lagrangian partial derivative to zero to obtain optimal resource allocation solutions for $\tau ,P_{i},P_{l,i}$.

The iteration process is provided in as follows:

$\Delta \lambda_{1}=\tau_{WEH}+\tau_{WIT}-1$
$\Delta \lambda_{2}=P_{i}-P_{i}^{max}$
$\Delta \lambda_{3}=P_{l,i}-P_{l,i}^{max}$
$\Delta \lambda_{4}=\left(P_{l,i}+P_{l,c}\right)\tau_{WIT}-\xi \sum_{i=1}^{I}P_{i}c_{i,l}\tau_{WEH}+e_{l.available}^{\left(\tau \right)}$

The initial Lagrangian multipliers are updated iteratively by $\lambda^{\left(t+1\right)}=\left(\lambda^{\left(t\right)}+\gamma^{\left(t\right)}\Delta \lambda \right)$ to obtain a new set of multipliers. This process is repeated until the optimal multipliers are realized when the proposed iteration algorithm reaches a point of saturation. Note that $\Delta \lambda =(\Delta \lambda_{1},\Delta \lambda_{2},\Delta \lambda_{3},\Delta \lambda_{4})$, and $\gamma^{\left(t\right)}$ is used to denote the step size of the iteration.

The process of computing optimal energy transfer time allocation; data transfer time allocation; and *l* device transmit power allocation, i.e., $\tau^{*}$, $P_{i}^{*}$, and $P_{l,i}^{*}$, is obtained by equating the Lagrangian partial derivative to zero, as follows:

By equating $\frac{\partial L}{\partial \tau_{WEH}}$, $\frac{\partial L}{\partial \tau_{WIT}}$, $\frac{\partial L}{\partial P_{i}}$, and $\frac{\partial L}{\partial P_{l,i}}$ to zero, the following optimal solutions can be determined:

(48) $$\begin{array}{cc} \frac{\partial L}{\partial \tau_{WEH}} & =\frac{B\sum_{l=1}^{L}}{In2}In\left(1+\frac{P_{l,i}d_{l,i}}{\sum_{l^{\prime }\geq l+1}P_{l^{\prime },i}d_{l^{\prime },i}+\sigma^{2}}\right)\\ & -r\cdot \sum_{i=1}^{I}(P_{i}+P_{c}-\sum_{k=1}^{K}\xi \left(c_{i,k}P_{i}\right)\\ & +\sum_{l=1}^{L}\xi \left(c_{i,l}Pi\right))+\lambda_{1}\left(WIT\right)\\ & +\lambda_{4}(\left(P_{l,i}+P_{l,c}\right)\tau_{WIT}\\ & -\xi \sum_{i=1}^{I}P_{i}c_{i,l}+e_{l.available}^{\left(\tau \right)}) \end{array}$$

(49) $$\begin{array}{cc} \frac{\partial L}{\partial \tau_{WIT}} & =\sum_{l=1}^{L}\left(P_{l,i}+P_{l,c}\right)+\lambda_{1}\left(\tau_{WEH}\right)\\ & +\lambda_{4}(\left(P_{l,i}+P_{l,c}\right) \end{array}$$

(50) $$\begin{array}{cc} \frac{\partial L}{\partial P_{i}} & =r\cdot \sum_{i=1}^{I}(1+P_{c}-\sum_{k=1}^{K}\xi \left(c_{i,k}\right)\\ & +\sum_{l=1}^{L}\xi \left(c_{i,l}\right))\tau_{WEH}\\ & -\lambda_{2}\left(1-P_{i}^{max}\right)+\lambda_{4}(\xi \sum {i=1}^{I}c_{i,l}\\ & +\tau_{WEH}+e_{l\cdot available}^{\left(\tau \right)}) \end{array}$$

(51) $$\begin{array}{cc} \frac{\partial L}{\partial P_{l,i}} & =\frac{B\sum_{l=1}^{L}\tau_{WEH}}{In2}\\ & \left(\frac{\sum_{l^{\prime }\geq l+1}P_{l^{\prime },i}d_{l^{\prime },i}+\sigma^{2}}{\sum_{l^{\prime }\geq l+1}P_{l^{\prime },i}d_{l^{\prime },i}+\sigma^{2}+P_{l,i}d_{l,i}}\right)\cdot \\ & \left(\frac{d_{l,i}}{\sum_{l^{\prime }\geq l+1}P_{l^{\prime },i}d_{l^{\prime },i}+\sigma^{2}}\right)+\\ & \sum_{l=1}^{L}\left(1+P_{l,c}\right)\tau_{WIT}+\lambda_{3}\left(1-P_{l,i}^{max}\right)\;+\\ & \lambda_{4}\left(1+P_{l,c}\right)\tau_{WIT} \end{array}$$

(52) $$\begin{array}{c} \frac{\partial L}{\partial \lambda_{1}}=\tau_{WEH}+\tau_{WIT}-1 \end{array}$$

(53) $$\begin{array}{c} \frac{\partial L}{\partial \lambda_{2}}=P_{i}-P_{i}^{max} \end{array}$$

(54) $$\begin{array}{c} \frac{\partial L}{\partial \lambda_{3}}=P_{l,i}-P_{l,i}^{max} \end{array}$$

(55) $$\begin{array}{cc} \frac{\partial L}{\partial \lambda_{4}} & =(\left(P_{l,i}+P_{l,c}\right)\tau_{WIT}\;-\\ & \xi \sum_{i=1}^{I}P_{i}c_{i,l}\tau_{WEH}e_{l\cdot available}^{\left(\tau \right)} \end{array}$$

The resource allocation algorithm for solving the optimization problems in (33) and (35) is presented in Algorithm 1.

To obtain optimal power and time resource allocations in each cycle of the proposed system, a Dinkelbach-based iteration algorithm is proposed and presented in Algorithm 2.

### 4.3. Dynamic HAP Resource Allocation Algorithm

This section presents the resource allocation algorithm (i.e., Algorithm 3) employed by the proposed system for allocating HAP resources. During the WIT phase, the HAPs are dynamically allocated to the *K* and *L* devices for data collection during a scheduled period to manage the energy consumption of the devices. This is achieved by exploiting the channel gain differences between the devices and the HAPs. This concept helps improve the communication channel quality of devices with a power channel gain to reduce the power used by the devices to report their individual data to the HAPs.
**Algorithm 3** HAP allocation in the WIT Phase**Require:** 
$\{m_{1},m_{2},\ldots ,m_{K}\}\;IoT\;devices\;$, $\left\{n_{1},n_{2},\ldots ,n_{L}\right\}\;IoT\;devices,\;and\;{\left\{s_{i}\right\}}_{i=1}^{2}$  1:At timeslot τ, compute the Euclidean distance between each *k* IoT device and ${\left\{s_{i}\right\}}_{i=1}^{2}$  2:Based on step (1), allocate each *k* IoT device to the nearest HAP $i\;\in \;{\left\{s_{i}\right\}}_{i=1}^{2}$  3:At the next timeslot, τ+1, compute the Euclidean distance between each *l* IoT device and ${\left\{s_{i}\right\}}_{i=1}^{2}$  4:Based on step (3), allocate each *l* IoT device to the nearest HAP $i\;\in \;{\left\{s_{i}\right\}}_{i=1}^{2}$

## 5. Results and Discussions

In this section, the performance of the proposed system is evaluated based on the baseline method in [26]. Consequently, the same simulation parameters as in [26] were also assumed in this study for comparison and validation purposes. The simulation parameters used in the experiments are presented in Table 4.

**Figure 3:**
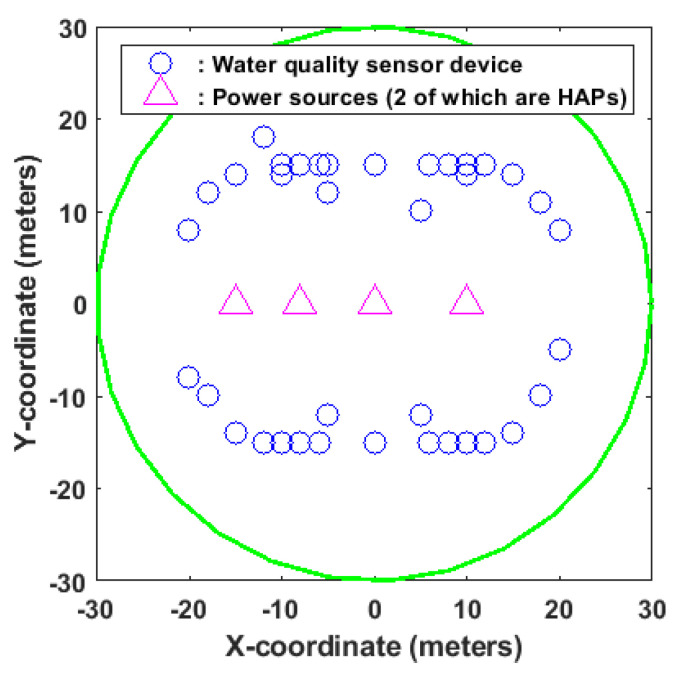
Network deployment.

The proposed system comprises two sequential groups of class A and B networks. The class A network is configured with a set of *K* water quality sensor devices, whereas the B network is configured with *L* water quality sensor devices. These devices are deployed within the communication coverage of the power sources and data collection devices, as shown in Figure 3.

### 5.1. Performance Comparison of Different Methods

**Figure 4:**
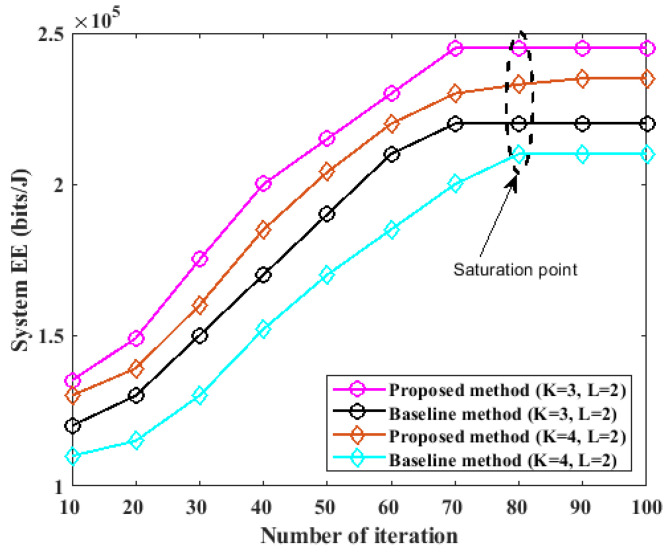
Performance comparison of the proposed method.

In this section, the proposed method is simulated and compared with a baseline method [26] to evaluate its performance. Similar to the baseline method [26], we consider a WPCN system with five sensor devices, six sensor devices, and three power sources. Consequently, two configurations were considered in the experiments. The first configuration included K=3, L=2, and I=3. The second configuration included K=4, L=2, and I=3. Two *I* devices were enabled to transfer power and collect water data from the devices. In both configurations 1 and 2, the *K* and *L* devices were enabled to concurrently perform energy harvesting within the period of $\tau_{WEH}$ using the proposed NOMA protocol. Using the proposed sequential strategy, only the class A network with *A* devices was enabled to perform water data transmission to the allocated HAPs within the period of $\tau_{WIT}$ since the water data of the *K* devices are more critical than those of the *L* devices. The proposed algorithm was enabled to simulate the proposed method, and it was disabled for the baseline method. The proposed method was simulated over a different number of runs (or iterations). In each run, the performance of the proposed method was compared with the baseline method, and the outcome of each iteration is presented in Figure 4.

From Figure 4, it can be deduced that both the proposed method and the existing method converged well to an optimal saturation point at approximately 80 runs. However, the performance of the proposed system for the two configurations considered validates that the proposed system is more energy-efficient than the baseline method. The proposed method outperformed the baseline method by approximately 12.65% and 16.49% for configurations 1 and 2, respectively. The efficiency of the proposed method can be attributed to the computation of the optimal resource allocation for network devices using the proposed resource allocation algorithm.

### 5.2. Impact of Noise Power on Energy Efficiency

**Figure 5:**
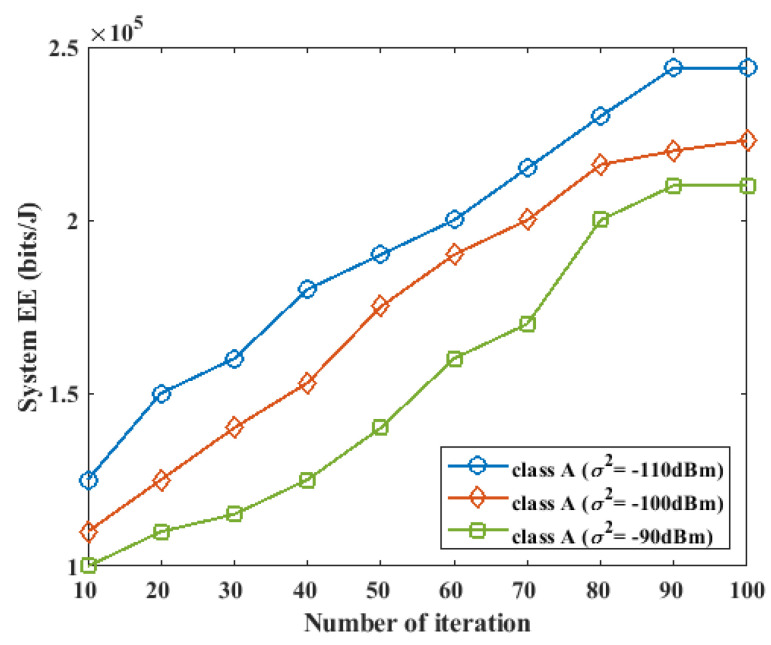
Impact of noise power on energy efficiency for class *A* device during the first phase of UL data transmission.

**Figure 6:**
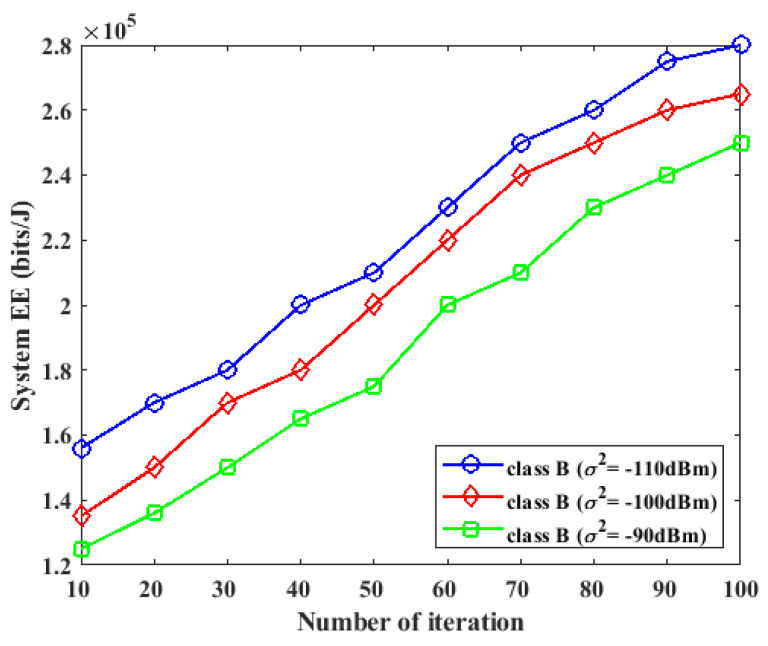
Impact of noise power on energy efficiency for class *B* device during the next phase of UL data transmission.

In this section, we investigate the effect of noise power on the performance of the proposed method. For this experiment, we considered a WPCN system with K=3 devices in the class A network, L=2 devices in the class B network, and I=3. The two classes of networks perform energy harvesting during the $\tau_{WEH}$ period. The class A devices are first enabled to perform data transmission in the current cycle and the B devices are scheduled to perform data transmission in the next cycle. The value of the system noise power was varied from −90 dBm to −110 dBm over a different number of iterations, and the energy efficiency performance of the system is presented in Figure 5 and Figure 6 for the two classes.

During the first cycle of the system, where the A devices transferred their data to the two HAPs in the system, as the noise power was varied from −90 dBm to −110 dBm, the energy efficiency of the system increased for a small value of noise power, while the energy efficiency decreased for a large value of noise power, as shown in Figure 5. During the next cycle of the system, where the B devices performed data transmission at the UL, the same effect during the first cycle of the system was also observed. The increase in the energy efficiency effect caused by a small value of noise power can be attributed to the increased data rate with low power consumption and low noise power.

### 5.3. Effect of the Number of Power Sources on Energy Efficiency

**Figure 7:**
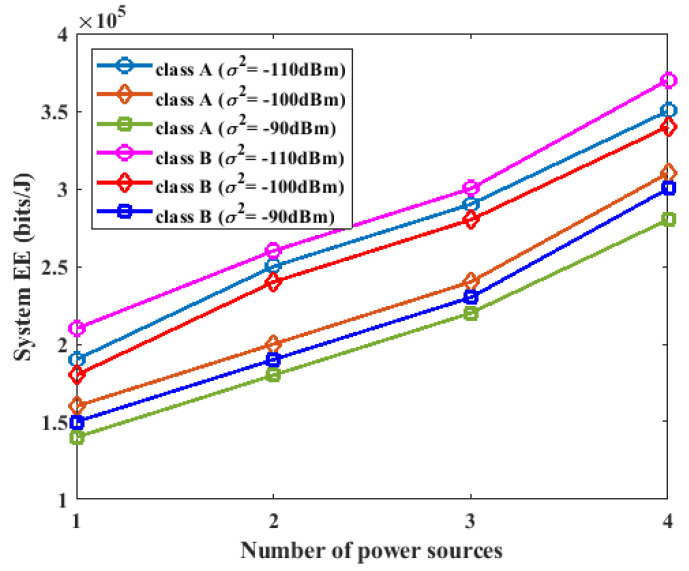
Impact of number of power sources on energy efficiency.

In this section, different numbers of power sources were used in the experiments to investigate the effect of the number of power sources on the energy efficiency of the proposed system. The proposed system was configured with K=3, L=2, $\sigma^{2}=-90$ dBm, $\sigma^{2}=-100$ dBm, and $\sigma^{2}=-110$ dBm, and the number of power sources was varied from one to five. From the results in Figure 7, it was observed that increasing the number of power sources resulted in increased energy efficiency of the system for both class A and class B devices. However, the class B devices achieved a higher energy efficiency than the class A devices. The class B devices achieved an increased energy efficiency because they had less data to transmit compared to the A devices. Hence, the class B devices spent less power on data transmission and were able to increase energy efficiency.

### 5.4. Impact of Sensor Device Transmit Power on Energy Efficiency

**Figure 8:**
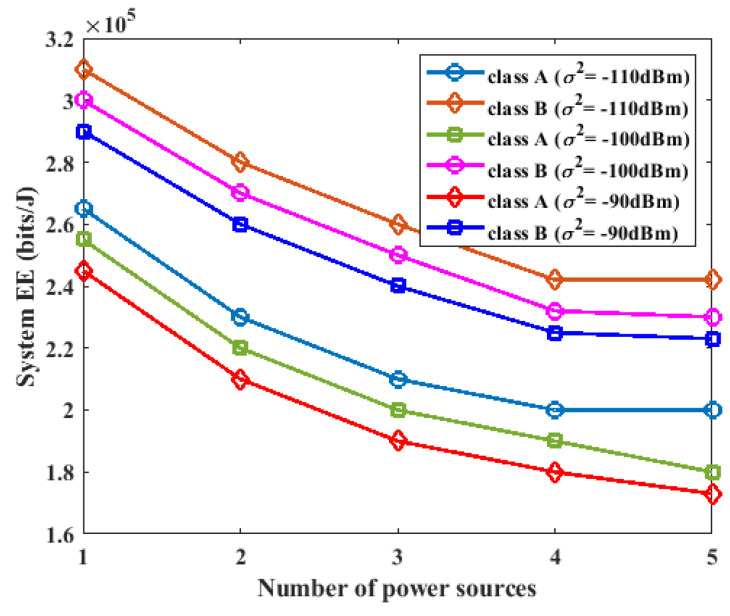
Impact of sensor device transmit power on energy efficiency.

In this section, we study the impact of different values of the sensor device transmission power on the energy of the proposed system. We consider K=3, L=2, I=3, $\sigma^{2}=-90$ dBm, $\sigma^{2}=-100$ dBm, and $\sigma^{2}=-110$ dBm for $P_{k,i}=P_{l,i}$ = 0.1 W, 0.2 W, 0.3 W, 0.4 W. The experimental results are shown in Figure 8. As shown in Figure 8, as the transmission power of the sensor devices in class A and class B was varied from 0.1 W to 0.4 W, there was a slight decrease in the energy efficiency of the system. This was a result of the trade-off effect between the total energy consumption and total throughput. When the transmission power of the sensor devices was increased, more data were supported by the system, and the energy consumption of the sensor devices increased. Consequently, the system energy efficiency decreased slightly.

### 5.5. Impact of QoS Data Requirements on the System EE

**Figure 9:**
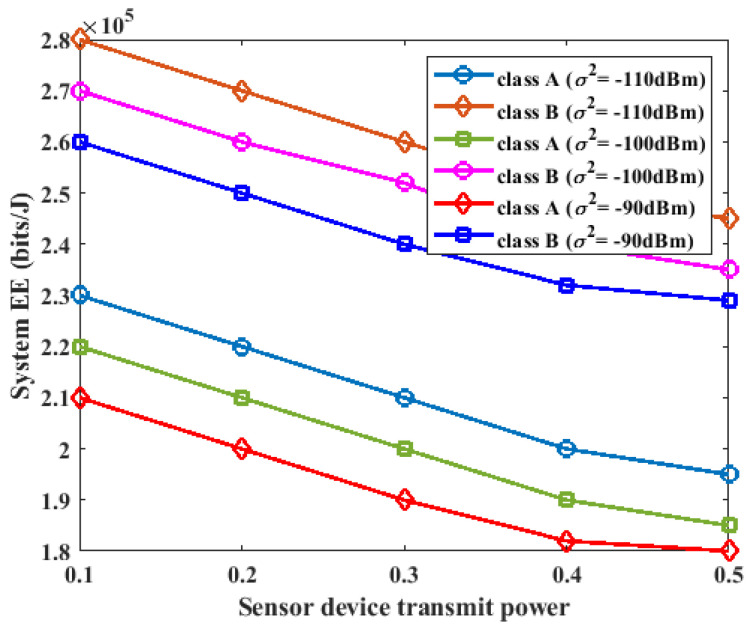
Impact of minimum QoS throughput on energy efficiency for the first configuration.

**Figure 10:**
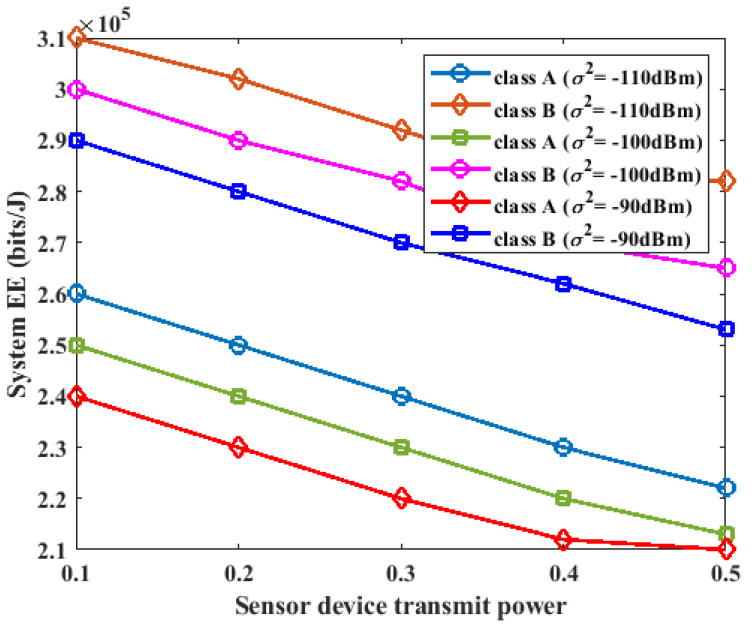
Impact of minimum QoS throughput on energy efficiency for the second configuration.

In this section, different values of the minimum QoS throughput requirements are investigated for the performance of the proposed system. In this experiment, two configurations were considered. The first system configuration included K=3, L=2, I=3, $\sigma^{2}=-90$ dBm, $\sigma^{2}=-100$ dBm, and $\sigma^{2}=-110$ dBm. The second system configuration contains K=3, L=2, I=4, $\sigma^{2}=-90$ dBm, $\sigma^{2}=-100$ dBm, and $\sigma^{2}=-110$ dBm The minimum QoS throughput requirement of the devices varied from 1000 bits to 5000 bits for the two system configurations. The experimental results for the two configurations are shown in Figure 9 and Figure 10.

## 6. Conclusions

IoT-enabled water quality monitoring systems are becoming increasingly popular due to their benefits over laboratory-based systems. However, these systems are resource-constrained, with limited power, computational, and bandwidth resources. As a result, they have drawn the attention of academics and practitioners to improve their performance. In this study, we introduced a multi-class communication strategy to classify the water quality sensor devices in the system. We designed a NOMA-based communication protocol to schedule and optimize the operation of the water quality sensor devices for energy harvesting and information transfer. We proposed a new resource allocation method to compute optimal power and time resource allocation for the devices. We also introduced a dynamic resource allocation method for hybrid access point (HAP) resource allocation for efficient data collection. Furthermore, we introduced edge computing into the water quality monitoring system proposed in this work to extend the traditional architecture based on cloud computing. This helps to improve the computational capacity of the system and enables local processing of water data at the water site to guarantee real-time water quality monitoring. Our proposed method outperformed an existing comparable baseline method by approximately 12.65% and 16.49% for two different configurations, demonstrating its effectiveness in improving the energy efficiency of a water quality monitoring system. In future studies, more research is required to explore ways to improve the energy efficiency of the wireless-powered water quality sensor devices used in water quality monitoring systems.

## Figures and Tables

**Table 1:** Comparison of the existing works with the proposed work.

Reference	Contribution of Related Works	Contribution of the Proposed Work
[20]	The authors designed a resource allocation algorithm to manage edge computation resource allocation in a network where all IoT devices participate in data transmission in the same cycle.	Unlike [20], we introduced a dynamic resource allocation method and an optimization-based method to jointly optimize energy harvesting and data transmission in a sequential multi-class WPCN, where each class of sensors operates sequentially to improve the overall system energy efficiency.
[21]	The authors designed a wireless- powered network where IoT devices perform complex tasks. Additionally, IoT devices can only send their data to a single base station.	Contrary to [21], we shifted complex tasks from IoT devices to reduce energy consumption. Additionally, we contributed a dynamic resource allocation method to optimally allocate multiple hybrid access points to improve system energy efficiency.
[22]	The authors designed a resource management scheme to offload computations in the network IoT devices concurrently.	Unlike [22], we introduced a sequential multi-class WPCN strategy for offloading computations in a sequential manner. Additionally, we contributed a dynamic resource allocation method to improve the overall system energy efficiency.
[25]	The authors designed a game theory-based resource allocation method to improve energy efficiency in cooperative network settings.	Unlike [25], we proposed a dynamic resource allocation method and an optimization-based method to jointly optimize the allocation of system resources to improve the overall system energy efficiency in sequential multi-class WPCN settings.
[26]	The authors designed a wireless- powered communication network with only one hybrid access point.	Different from [26], we contributed a sequential multi-class WPCN with dynamically allocated hybrid access points to improve energy efficiency.
[27]	The authors designed a wireless- powered cooperative IoT network where devices transmit data in the same cycle.	Different from [27], we contributed a sequential multi-class WPCN where devices transmit data in different cycles to improve energy efficiency.
[28]	The authors designed a wireless- powered communication network where IoT devices uses a multi-hop communication strategy to communi- cate with a single base station.	Contrary to [28], we contributed a sequential multi-class WPCN where IoT devices uses single-hop communication to communicate with dynamically allocated hybrid access points to improve energy efficiency.

**Table 2:** Project Requirements.

Requirement	Range
pH sensor	0–14
Conductivity sensor	100 μS/cm–200 mS/cm
*E. coli* sensor	1–1000 CFU/100 mL
Residual chlorine sensor	0–10 mg/L
Dissolved oxygen sensor	0–20 mg/L
Transmitter/HAP	1 W–3 W
Edge node	≤5 m from HAPs
ZigBee radio	Above 100 m

**Table 3:** List of acronyms.

Acronymn	Definition
IoT	Internet of Things
NOMA	Non-orthogonal multiple access
HAP	Hybrid access point
WEH	Wireless energy transfer
WIT	Wireless information transfer
$\tau_{WEH}\left(s\right)$	Time slot for wireless information transfer
$\tau_{WIT}\left(s\right)$	Time slot for wireless energy transfer
$c_{i,k}$ and $c_{i,l}$	Downlink communication channel gains
$d_{k,i}$ and $d_{l,i}$	Uplink communication channel gains
$P_{i}\left(W\right)$	HAP transmission power
SIC	Successive interference cancellation
B	System’s bandwidth
DL	Downlink
UL	Uplink
$HAPs\;{\left\{s_{i}\right\}}_{i=1}^{2}$	Power sources
$e_{k\cdot harvest}^{\tau }$	Energy harvested by *K* sensor devices
$e_{l\cdot harvest}^{\tau }\left(J\right)$	Energy harvested by *L* sensor devices
$e_{k\cdot transmit}^{\tau }\;\left(J\right)$	Energy used by each sensor device *k* for UL data transfer
$e_{l\cdot transmit}^{\tau }\;\left(J\right)$	Energy used by each sensor device *l* for UL data transfer

**Table 4:** Simulation Parameters.

Parameter	Setting	References
$P_{i}^{max}$	3 W	[26]
$P_{k,i}^{max}=P_{l,i}^{max}$	0.3 W	[26]
$P_{c}$	0.5 W	[26]
Frequency	2.4 GHz	[26]
ξ	0.9	[26]
$\sigma^{2}$	−110 dBm	[26]
$r_{k,i}=r_{l,i}$	2 kbit/s	[26]
τ	1 s	[26]
*B*	20 kHz	[26]

## Data Availability

Not applicable.
